# The role of microRNA-30a and downstream snail1 on the growth and metastasis of melanoma tumor 

**DOI:** 10.22038/IJBMS.2019.32317.7745

**Published:** 2019-05

**Authors:** Jahangir Noori, Shaghayegh Haghjooy Javanmard, Mohamadreza Sharifi

**Affiliations:** 1 Department of Physiology, Isfahan University of Medical Sciences, Isfahan, Iran; 2 Applied Physiology Research Center, Isfahan Cardiovascular Research Institute, Department of Physiology, School of Medicine, Isfahan University of Medical Sciences, Isfahan, Iran; 3 Department of Genetics and Molecular Biology- Isfahan University of Medical Sciences, Isfahan, Iran

**Keywords:** Epithelial-mesenchymal – transition, Melanoma, Metastasis, miR-30a, Neoplasm, Snail1

## Abstract

**Objective(s):**

Growing evidences have indicated microRNAs as modulators of tumor development and aggression. On the other hand, a phenomenon known as epithelial-mesenchymal transition (EMT) that indicates a transient phase from epithelial-like features to mesenchymal phenotype is a key player in tumor progression. In this study, we aimed to assess the potential impacts of miR-30a-5p as an inhibitor of melanoma progression and metastasis.

**Materials and Methods:**

MiR-30a-5p was transfected into B16-F10 melanoma cells. Then, the B16-F10 cells were injected subcutaneously or intravenously (IV) in to C57BL/6 mice. Then, the mice were euthanized and tumor size, tumor weight, snail1 protein expression and nodules in the lungs were evaluated.

**Results:**

The migration of cancerous cells was significantly suppressed in vitro following the ectopic presentation of miR-30a-5p into B16-F10 melanoma cells. Furthermore, the metastatic behavior of the neoplastic cells was further suppressed in a xenograft mouse model of melanoma as observed with limited lung infiltration. We also found that transfected miR-30a-5p into melanoma cells could decrease snail1 and N-cadherin expression.

**Conclusion:**

MiR-30a-5p may represent an effective therapeutic target for the management of melanoma and other snail-overexpressing neoplasms.

## Introduction

The post-transcriptional regulation of gene expression by small non-coding RNA (known as miRNAs) presents a viable mechanism by which cells regulate their functions. This essential function of miRNAs is warranted through targeting nearly 30% of cellular proteins (1).

The functional disruption of miRNAs network is an inevitable events for development of a variety of cancers including melanoma (2). Either downregulated or upregulated miRNAs can

 be potential therapeutic targets in cancers. This may be achievable by recruiting either agents with agonistic or antagonistic activities for downregulated and overexpressed miRNAs, respectively (3).

Melanoma, an aggressive skin neoplasm, constitutes the second most rapidly growing neoplasm in human after lung cancer. During the recent decade, the incidence rate of melanoma has increased by two-folds. However, there is currently no curative therapy available, particularly for its advanced stages. So, the underlying molecular pathways contributing to melanoma development and progression have been under intense investigations (4).

An extensively studied member of miR-30 family, miR-30a-5p, has previously been shown to be downregulated in multiple neoplasms (5-8). Studies demonstrating the dysregulation of miR-30a-5p in melanoma cancerous cells and tissues (9) suggest that miR-30a-5p can promote suppressive activity against tumor development in melanoma. Furthermore, miR-30a-5p downstream targets have been indicated as the 3′ UTR of Snail. Moreover, miR-30a-5p is associated with suppressed epithelial to mesenchymal transition (EMT), a major contributor to metastatic and progressive behaviour of neoplastic cells, (10) in non-small cell lung cancer (NSCLC) cell lines.

Snail is considered to be one of the most potent transcriptional factor contributing to the EMT in neoplastic cells, which inhibits E-cadherin expression (11).

Despite studies in multiple cancers, the role of miR-30a-5p as a potential modulator of downstream events leading to melanoma tumor progress and metastasis is unknown. In this study, we aimed to investigate the potential suppressive capacity of miR-30a-5p against melanoma progression. As well, we assessed if modulation of miR-30a-5p could block pulmonary metastasis of melanoma cancerous cells. We further investigated if miR-30a-5p could recruit snail or E-cadherin as potential molecular adaptors.

## Materials and Methods

***Cell culture and Transfection***

The National Cell bank of Iran (NCBI, Pasteur institute of Iran) provided the B16-F10 melanoma cells. The cells were then cultured in DMEM medium. For supplementation, L-glutamine (4 mM), glucose (4.5 g/l) and fetal bovine serum (FBS; 10%) were added to the medium. The cells were subsequently incubated in 5% CO_2_ at 37 ^°^C. 

The miR-30a-5p mimics and scramble were purchased from Bioneer. The sequence of mimics (miR-30a-5p m) was 5′-UGUAAACAUCCUCGACUGGAAG-3′. On the other hand, the sequence of scramble miRNA (scr ODN) was 5′-CAGUACUUUUGUGUAGUACAA-3′. By applying Lipofectamine 2000 according to the instructions mentioned by the provider (Invitrogen, USA), the melanoma cells were transfected with 50 pmol/µlL oligonucleotides at 80% confluence. For confirming the cellular uptake assay, FAM-miR-30a-5p (50 nM) was used and then flowcytometry analysis was performed. B16-F10 cells were cultured in 12-well plates. The cell density was adjusted as 2 × 10^5^ cells per well. The cells were incubated in the mentioned condition for 24 hr at 37 ^°^C in 5 % CO_2_.

Following 24 hr of the transfection, the cells were diluted in phosphate-buffered saline (PBS). After that, flowcytometry analysis (FACSCalibur, BD Biosciences, USA) was carried out to determine the rate of FAM-miR-30a-5p cellular uptake in triplicate. 

The cells were divided into 3 groups, mimic, scramble and non-treated controls. Total RNA was extracted 48 hr after transfection.

***Cell viability***

The 3-(4, 5-dimethylthiazol-2-yl)-2, 5- diphenyltetrazolium bromide (MTT) assay was applied for evaluating cell viability. Either non-transfected (negative control) or miR-30-5p transfected cells seeded in 96-well plates (2×10^4^ cells per well) were incubated with 0.2 mg/ml MTT (Sigma-Aldrich, Germany) for 24 hr. Then, the plates were kept in 37 °C for 4 hr. Finally, 200 µl dimethyl sulfoxide (Sigma-Aldrich) was added to the wells to remove the culture medium replacement. The optical density was read for determining cell viability.

***Cell migration assay***

The migration potency of neoplastic cells was determined utilizing uncoated membranes in transwell inserts (8-μm pore size) (SPL, Life Sciences, Korea). The upper chamber was loaded with either the suspensions (in serum-free medium) of untreated (control) or transfected cells (3×10^4^). This is while the mixture of medium (500 µl) in 10% FBS was applied in the lower chamber as a chemoattractant. Non-migratory cells were removed from the top membrane after 12 hr of incubation in the chamber. On the other hand, migrating cells were characterized with crystal violet staining (0.05% w/v) and enumerated under 5 random microscopic fields. All the procedures were conducted in triplicates.

***Subcutaneous and pulmonary metastasis mouse model***

Ethical considerations were followed during the experiment. Our study was also approved by the Ethics Committee of Isfahan University of Medical Sciences. All animal methods were conducted following the guidelines of the National Institutes of Health Guide for the Care and Use of Laboratory Animals.

C57BL/6 male mice (4-6 weeks) were purchased from the Pasteur institute of Iran (Tehran, Iran). In order to acclimatization to the environment, the mice were housed 2 weeks before experiment in a sterile condition, under 12 hr light/dark intervals, at room temperature and free access to water and food pellets. 

Either miR-30a-5p mimic or scramble was transfected to the B16-F10 cells (the volume of 50 pmol/µl) following 24 hr of seeding. After 24 hr, 1 × 10^6^ cells (from mimic, scramble and non-treated controls) resuspended in 100 μl sterile PBS and were subcutaneously administrated (at the right flank) or IV via tail vein (n=6 per group, the day zero). The tumor masses were observable at the day 8-9. The mice were killed at the day 20 after inoculation. For quantifying pulmonary black nodules, the lung tissues were obtained, then washed (PBS) and finally fixed (using neutral-buffered formaldehyde).

**Figure 1:**
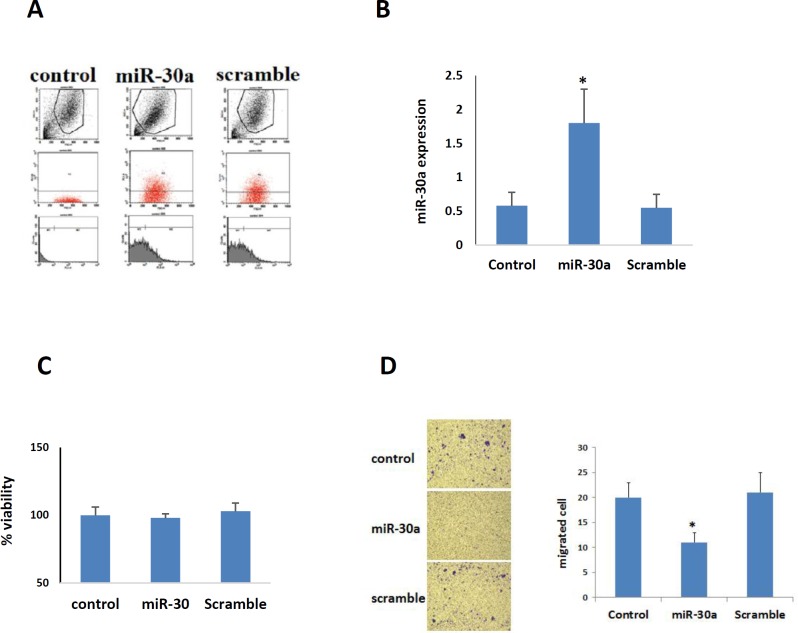
A. Evaluation of transfected melanoma cell with flow cytometry; over 75% of cells have been transfected by miR-30a-5p and scramble. B. Real-time PCR of miR-3a-5p in B16F10 melanoma cell lines after transfection with miR-30a-5p and scramble in comparison with control. C. MTT analysis showed that both miR-30a-5p and scramble have no obvious effects on viability of melanoma cells. D. Image and histogram of cell migration evaluated by Boyden chamber.*, *P*<0.05 compared to the control and scramble groups

**Figure 2:**
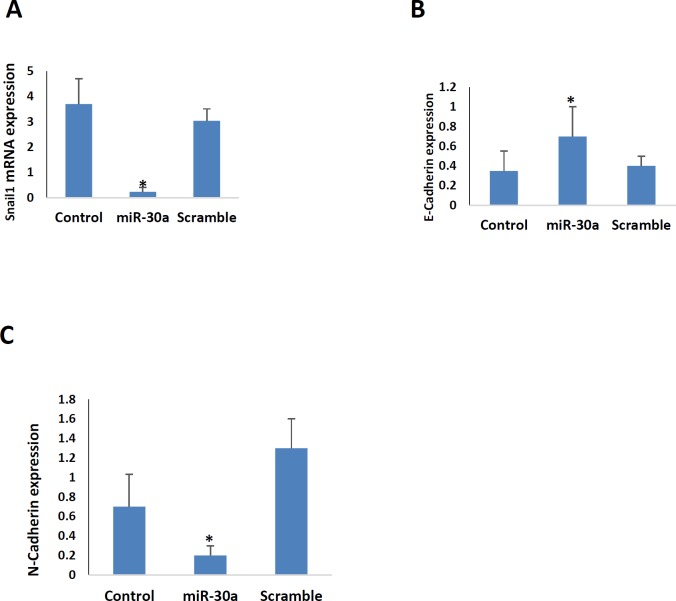
Real-time PCR was performed to measure relative expression levels of A. snail1, B. E-cadherin and C. N-cadherin in B16F10 melanoma cell lines after transfection with miR-30a-5p and scramble in comparison with control. *,* P*<0.05 compared to the control and scramble groups

**Figure 3:**
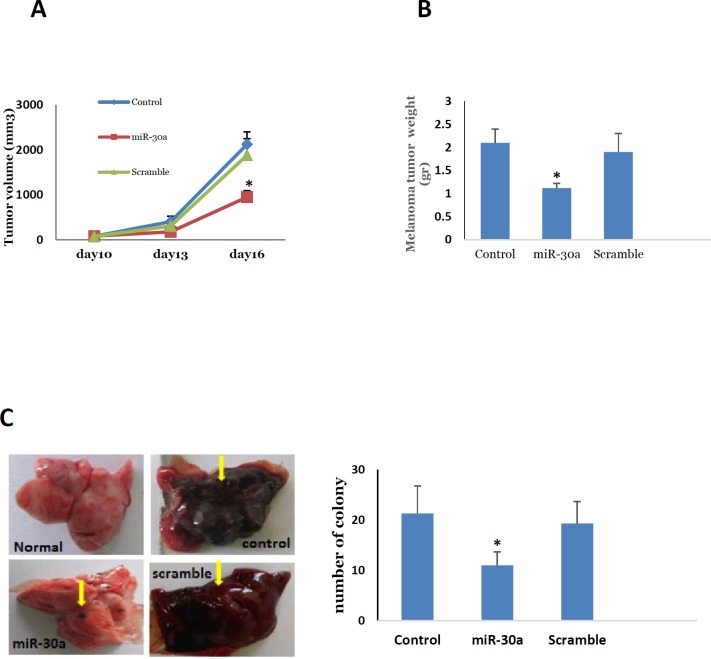
Comparisons of melanoma A. tumor size, B. tumor weight, C. number of lung colony among the control, miR-30a-5p and scramble groups. Note: * *P*<0.05 compared to the control and scramble groups

**Figure 4:**
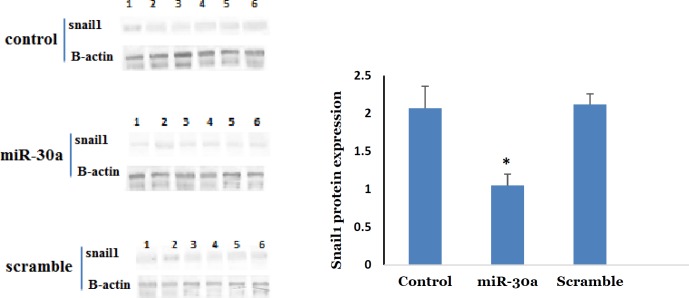
Comparisons of A. Western blot expression of snail1 protein in melanoma tumor, among the control, miR-30a-5p and scramble groups. Note: * P<0.05 compared to the control and scramble groups

**Table 1:** The sequences of specific forward (F) and reverse (R) primers used to amplify the studied genes

**Gene**		**Sequence (5′ - 3′)**
Snail1	F	GCGTGTGTGGAGTTCACCTT
	R	CCAGGAGAGAGTCCCAGATGAG
GAPDH	F	TGGAGAAACCTGCCAAGTATGATG
	R	AGTGGGAGTTGCTGTTGAAGTC
E-Cadherin	F	TCCTCGCCCTGCTGATTCTG
	R	CTGGTCTTCTTCTCCACCTCCTTC
N-Cadherin	F	CGCCATCATCGCTATCCTTCTGT
	R	GGCTCAAGTCATAGTCCTCCTGGTCTT

***Quantitative RT-PCR for assessing miRNA expression***

The miRCURY LNA Universal RT miRNA PCR system was applied following the manufacturer’s instructions to detect miRNA in the neoplastic transfected cells (SYBR green, Applied Biosystems 7900HT real-time PCR). For internal control of the RT-PCR reaction, an RNA spike-in was adjoined to the transfected sequence prior to the synthesis of respective cDNA. The small nuclear U6 RNA was recruited for normalizing the data. Relative miRNA expression was determined by calculating the mean difference between the cycles thresholds (CT) that were recorded for the reactions. The differences between the mean CTs of the normalized RNA (i.e. U6 small nuclear RNA) and miR30-5p in each sample were calculated and represented as ΔCT. The ΔCTs were expressed as the mean fold change respective to the mean of the control (i.e. delivering 2−ΔΔCT as a representative of the expression level of the interest miRNA). The specific primers were applied as miRNA primer set (mmu-miR-30a-5p: MIMAT0000128, targeted sequence: 5′-UGUAAACAUCCUCGACUGGAAG-3). Primer set for 5s ribosomal RNA (rRNA) and an internal control was used as control. All the reactions were performed in triplicate.

***Western blot***

Melanoma tumor cells were initially degraded on ice-cold radioimmunoprecipitation assay (RIPA) buffer (137 mM NaCl, 20 mM Tris-HCl, pH 7.5, 0.5 mM sodium orthovanadate, 1 mM phenylmethylsulfonylfluride, 1 g/ml leupeptin, 10 g/ml aprotinin) containing protease inhibitor by homogenizer. The supernatant was separated by a 30-min centrifugation at 13,000 g. Then, the Bradford protein assay kit (Bio-Rad) was applied for determining protein concentrations. After boiling the samples in the sample buffer (1:1 ratio, 5 min), the protein contents of the lysates were separated by 12% SDS-PAGE gel. After electrophoresis, the protein bands on the gels were transferred onto a PVDF membrane at 80 volt for 90 min. The blocking phase was then performed by using 5% nonfat milk in Tris-buffered saline (TBS; 1 hr., room temperature). The membranes were washed in the mixture of PBS and 0.2% Tween-20, and incubated. After this stage, the primary mouse antibody (monoclonal, 1/1,000) 1 hr was added. Antibodies against SNAIL (H-130) and β-actin were purchased from santacruz (Santa Cruz Biotechnology, Inc., CA, USA). After another washing phase in the previously mentioned washing solution, the secondary antibody (mouse anti -goat IgG-HRP, Santa Cruz Biotechnology) with the final concentration of 1:5,000 was applied. The incubation continued at room temperature for another 60 min. Finally, the membranes were again washed with the washing solution and the reaction was developed with 0.05% (w/v) of 3, 3′-diaminobenzidine (DAB) and 0.3% H_2_O_2_ in PBS.

***Statistical analysis***

Data are shown as the mean ± SD. The analyses were performed in SPSS 18 software (SPSS Inc., Chicago, IL, USA). One-way analysis of variance was used for between groups comparisons. The *P* value < 0.05 was regarded as significance cut off. One-way ANOVA and Mann-Whitney U tests for nonparametric analyses were performed for multiple group comparisons of the data. The *post hoc* Tukey test was further applied to detect any significant differences between the mean values.

## Results

***The effect of MiR-30a-5p suppression on migration of B16F10 melanoma cells***

Decreased expression of miR-30a-5p was found in melanoma cells compared to negative controls (12). The efficiency of miR-30a-5p oligonucleotides transfection was here confirmed by both flowcytometry and qPCR (Figure 1A, 1B). The proliferative activity of the transfected neoplastic cells did not show any significant alternation, as revealed by MTT assay (Figure 1C). However, the transfected cancerous cells represented a significant reduction in both migration and motility activities, as shown by transwell assays (*P*<0.05, Figure 1D).

***The expression of snail, E-cadherin and N-cadherin in miR-30a-5p transfected B16F10 cells***

The snail, E-cadherin, and N-cadherin are indicators of the EMT phenomenon (their primers were shown in Table 1). This process is an inevitable route in tumor progression. As shown in Figure 2A, 2B and 2C, the expression of snail, and N-cadherin were downregulated in miR-30a-5p transfected B16F10 cells. This is while the expression of E-cadherin increased in these cells. 

***MiR-30a-5p suppressed melanoma tumor growth and metastasis in vivo***

For assessing melanoma tumor growth in the miR-30a-5p transfected cells, the melanoma tumor was developed at subcutaneous. We observed that tumor growth was significantly lower in miR-30a-5p cancer cells in comparison with non-transfected cells (Figure 3A, *P*<0.05). As revealed by the tumor weight at the day 16 after tumor induction, the transfected mice revealed a significantly lower tumor weight respective to control group (Figure 3B, *P*<0.05). This was accompanied with a significant reduction in the expression of snail in tumor tissue of miR-30a-5p treated group (Figure 4, *P*<0.05). Based on these observations, one can conclude that the upregulation of miR-30a-5p may be potent route to prevent tumor growth in melanoma. Furthermore, C57BL/6 mice that received 30a-5p transfected cells were used to evaluate the metastatic behavior of neoplastic cells to the lungs. For this, the transfected cells were intravascularly entered into the mice body by the tail. Histological examination of pulmonary tissues was performed 20 days following tumor cell induction. The metastatic nodules were enumerated, which revealed a significant decrease in themiR-30a-5p administrated mice respective to control (Figure 3c, *P*<0.05). This indicates a dramatic suppressing effect on metastatic features of miR-30a-5p transfected melanoma cells suggesting this miRNA as a potential negative modulator of the invasive behavior of B16F10 melanoma cells both *in vitro* and *in vivo*. 

## Discussion

Progression and recurrence of cancers are main contributors to cancer-associated mortality worldwide. The EMT process is a critical phenomenon facilitating tumor progression and metastasis. MicroRNAs, as small non-coding RNAs, have been suggested as modulators of EMT process in neoplastic cells. The EMT process itself constitutes to a change in phenotypic features of neoplastic cells delivering them more invasive potential. On the other hand, miRNAs can modify phenotypic characteristics of melanoma cells by altering gene expression patterns and activating specific transcription machineries within cells. In this way, miRNAs can contribute to EMT phenomenon. The miR-30a-5p has been noted to be downregulated in melanoma compared to normal controls (12). It has been demonstrated that activated BRAF can downregulate several miRNAs including miR-let7i, miR-10, miR-22, miR-26a, miR-30, miR-34, miR-125a, and miR-211 (13).

Our results showed that miR-30a-5p has no toxic effect on B16F10 cells in 24 hr, while it has a potent suppressor effect on melanoma tumor growth *in vivo*. Indeed, the progression of tumor volume *in vivo *has not straightforward correlation with cell proliferation rate *in vitro*. Beside proliferative potential, tumor growth is also affected by apoptosis (i.e. higher apoptosis rate rendering lower tumor growth capability) (14). It has been noted that overexpression of miR-30a -5p induces apoptosis in neoplastic cells (10, 15, 16). All the above-mentioned studies have shown the anti-proliferative as well as apoptosis inducing effect of miR-30a-5p at least 48 hr after miR-30a-5p transfection. So, we just want to show that the same number of viable B16F10 cells was injected to the mice 24 hr after transfection. 

On the other hand and in consistent with our results, other studies reported no significant alternations in the proliferative and sphere forming indices after increased miR-30a-5p expression (17). miR-30a-5p may be of critical importance in dedicating stem-like features to several cancer cell lines (18-21). Decreased melanoma tumor growth *in vivo* might be explained by decreased numbers of cancer stem cells in miR-30a -5p melanoma cells. Tumor suppressive capability of miR-30a-5p has been described in multiple human cancers (such as colon, lung, breast, prostate, thyroid, liver and gastric). Unlike these, miR-30-5p represented oncogenic properties in some other neoplastic conditions such as glioma (22). Therefore, miR-30a-5p may present dual roles (either tumor suppressor or inducer) in the pathogenesis of different cancers depending on the tissue origin. In both *in vitro* and *in vivo* observations in present study, the beneficial impacts were established for miR-30a-5p in the suppression of migration, invasion, metastasis and tumor growth of transfected melanoma cells. Moreover, all these events were associated with suppressed SNAIL1 expression, functional target of miR-30a-5p. Snail1 has been suggested as contributor to tumor metastasis through EMT phenomenon. Accordingly, our findings propose miR-30a-5p as a main underlying adaptor progression and metastasis of melanoma cells through EMT process. miR-30-5p family has been associated with reduced Snail1 expression in cancers. In fact, these miRNAs have been downregulated in metastatic cancers suggesting miR-30 as a promising therapeutic target in cancer.

In agreement with our results, large-scale miRNA expression arrays have revealed lower expression of miR-30 in multiple human neoplastic cells such as colon, lung, breast, prostate, thyroid, acute myeloid leukemia and liver (23-28) as well as bladder cancer, colorectal cancer, ovarian cancer and esophageal squamous cell carcinoma (29). Furthermore, miR-30a-5p has been found to reduce E-cadherin expression and suppress EMT process (8), and to be more frequently downregulated in several other metastatic cancers (6). However, we here reported for the first time a critical role for miR-30a-5p as a potential therapeutic target in melanoma.

It has been reported that miR-30a-5p can target snail1 pathway to suppress transforming growth factor-beta (TGF-β) activity. According to this finding, it has been shown that reactivating miR-30a-5p could suppress EMT phenomenon by downregulating SNAIL1 in NSCLC cell lines (6).

As mentioned before, miR-30a-5p has been noted as a main modulator of EMT process in cancerous cells. This process can be associated with altered expression of some extracellular components such as matrix metalloproteinase-3 (MMP3) and vimentin. On the other hand, reports have shown that lower expression of miR-30a-3p is correlated with lower levels of MMP3 and vimentin as well as higher levels of E-cadherin in hepatocellular carcinoma cells (29).

A recent study showed the effect of miR-30a-5p on biological function of astrocyte elevated gene 1 (AEG-1) in hepatocellular carcinoma (HCC) cell lines. The result of the recent report suggest the AEG-1 as a potential target of miR-30a-5p in HCC(15). Through activating multiple downstream signaling pathways (Wnt/β-catenin, mitogen-activated protein kinase (MAPK), nuclear factor (NF)-κB, and phosphoinositide-3-kinase/ protein kinase B (PI3K/Akt)), AEG-1 may be involved in tumor progression.

Besides, it has been reported that another molecule, metadherin, was negatively correlated with the levels of miR-30a-5p in hepatic cancer suggesting the transcript of this molecule as another potential target of miR-30a-5p. Furthermore, upregulation of miR-30a-5p in HCC cells was accompanied with lower proliferation rate and colony formation of neoplastic cells (30). These were in parallel with the findings of Zhang *et al.* who showed suppressive effects of miR-30a-5p on tumor progression and metastasis in breast cancer in part through targeting metadherin (31). Furthermore, lower expression of vimentin that was correlated with overexpressed miR-30a-5p inhibited breast tumor progression in another study (32).

Also, it has been demonstrated that miR-30a-5p can target the phosphatidylinositol 3 kinase regulatory subunit resulting in downregulated protein, and thus inhibiting the invasion and metastasis (33). 

Recent findings have also demonstrated the role of miR-30a-5p in suppression of colon cancer progression by the inhibition of integrin β3 (34).

## Conclusion

We here for the first time demonstrated that miR-30a-5p suppressed melanoma tumor growth and metastasis. These observations support miR-30a-5p as a tumor suppressor. Furthermore, miR-30a-5p may represent an effective therapeutic target in melanoma and other cancers overexpressing snail. 

## Data Availability

All data generated or analyzed during this study are included in this article and is available.
